# Carboxyl-terminal fusion of E7 into Flagellin shifts TLR5 activation to NLRC4/NAIP5 activation and induces TLR5-independent anti-tumor immunity

**DOI:** 10.1038/srep24199

**Published:** 2016-04-11

**Authors:** Kuo-Hsing Lin, Li-Sheng Chang, Chun-Yuan Tian, Yi-Chen Yeh, Yu-Jie Chen, Tsung-Hsien Chuang, Shih-Jen Liu, Chih-Hsiang Leng

**Affiliations:** 1 National Institute of Infectious Diseases and Vaccinology, National Health Research Institutes, Zhunan Town, Miaoli 350, Taiwan ROC; 2 Institute of Biotechnology and Department of Life Science, National Tsing-Hua University, Hsinchu 305, Taiwan ROC; 3 Immunology Research Center, National Health Research Institutes, Zhunan Town, Miaoli 350, Taiwan ROC; 4 Graduate Institute of Immunology, China Medical University, Taichung 402, Taiwan ROC

## Abstract

Flagellin has the capacity to activate both Toll-like receptor 5 (TLR5) and Nod-like receptor C4 (NLRC4)/neuronal apoptosis inhibitory protein 5 (NAIP5) inflammasome signaling. We fused E7m (the inactivated E7 of human papillomavirus) to either end of the flagellin protein, and the resulting recombinant flagellin-E7m proteins (rFliCE7m and rE7mFliC) were used as immunogens. Both fusion proteins activated receptor signaling to different degrees. rE7mFliC-induced TLR5 activity was 10-fold higher than that of rFliCE7m, whereas rFliCE7m activated the NLRC4/NAIP5 pathway more strongly. Therefore, these recombinant proteins provided a tool to investigate which signaling pathway is critical for the induction of antigen-specific T cell responses and anti-tumor immunity. We demonstrated that rFliCE7m induced higher levels of E7-specific IFN-gamma-secreting cells and cytotoxic T lymphocytes (CTLs) than rE7mFliC, and a single injection with rFliCE7m but not rE7mFliC inhibited E7-expressing tumor growth *in vivo*. Furthermore, we confirmed that CD8^+^ T cells played a major role in the anti-tumor immunity induced by rFliCE7m. These findings suggested that the NLRC4/NAIP5 intracellular signaling pathway was critical for the induction of anti-tumor immunity. These observations provide important information for the rational design of flagellin-based immunotherapy.

Flagellin is the monomeric subunit of bacterial flagella and a well-known pathogen-associated molecular pattern (PAMP) protein. Flagellin has the capacity to regulate immune responses by activating epithelial cells, endothelial cells, T cells, dendritic cells (DCs), macrophages and neutrophils^1^,^2^,^3^,^4^,^5^,^6^. The receptors that recognize flagellin have been identified and include membrane-bound Toll-like receptor 5 (TLR5)^7^,^8^ and cytosolic nucleotide oligomerization domain (NOD)-like receptors (NLRs)^4^. Binding to TLR5 can trigger DCs or other antigen-presenting cells to secrete proinflammatory cytokines and induce adaptive immune responses^5^. After entering the cytosol, flagellin interacts with the neuronal apoptosis inhibitory protein 5 (NAIP5) to stimulate the NLR family and caspase activation recruitment domain (CARD)-containing 4 (NLRC4) for inflammasome activation^9^,^10^,^11^,^12^. The NLRC4/NAIP5 inflammasome activates cysteine protease caspase-1, resulting in the secretion of the proinflammatory cytokines interleukin-1β (IL-1β) and IL-18 from innate cells. The inflammasome can also induce a rapid inflammatory cell death known as pyroptosis to protect from pathogen infection^4^,^13^. Additionally, a number of studies have demonstrated that flagellin promotes cytokine production by a range of innate cell types and triggers the recruitment of T lymphocytes to the lymph node. Moreover, flagellin promotes strong antigen (Ag)-specific CD4^+^ T^14^ and CD8^+^ T cell responses^15^ through the direct stimulation of TLR5-expressing CD11c^+^ cells^16^. Based on its ability to trigger innate and adaptive immune responses, flagellin is considered a potent adjuvant for vaccines and immunotherapy^17^,^18^,^19^,^20^.

Flagellin consists of four domains designated D0, D1, D2 and D3 that resemble an upper case Greek letter gamma (Γ). The N-terminal chain starts at D0, extends through D1 and D2 to reach D3 and then returns through D2 and D1, with the C-terminal chain ending in D0^21^. The D1 domain directly interacts with TLR5^22^, and both the N- and C-termini of flagellin are required for TLR5 activation^23^. However, the interaction of flagellin with NAIP5 occurs through the 35 C-terminal amino acids of flagellin^24^. Although many flagellin-fused antigens have been demonstrated to be effective vaccines in animals^25^,^26^,^27^, whether the position of the antigen within flagellin affects the activation of TLR5 or the NLRC4/NAIP5 inflammasome is unclear.

Flagellin fusion proteins are effective vaccine candidates that induce protective antibody responses against microbial challenges in animal models^18^. However, there have been limited studies on the induction of CD8^+^ CTL responses against tumor growth using flagellin fusion proteins. In this report, we fused the inactivated E7 (rE7m) domain of HPV16 with flagellin (rFliC) derived from *Salmonella enterica* serovar Typhimurium to either the N- or C-terminus to produce recombinant rE7mFliC and rFliCE7m proteins. We compared activation of TLR5 and the NLRC4 inflammasome by these two flagellin fusion proteins, and the anti-tumor immunity of these proteins was evaluated using a mouse cancer model. We discovered that rE7mFliC induced higher levels of TLR5 activity than rFliCE7m, whereas rFliCE7m was more effective at inducing NLRC4 inflammation activity. Furthermore, rFliCE7m induced stronger cellular immune responses and anti-tumor immunity than rE7mFliC. Clearly, the fusion of flagellin to the immunogen at different termini showed a bias in signaling pathway activation and affected the induction levels of anti-tumor immunity. This finding is crucial for the development of flagellin-based immunotherapies.

## Results

### Production and characterization of recombinant flagellin and flagellin fusion proteins

**Figure 1:**
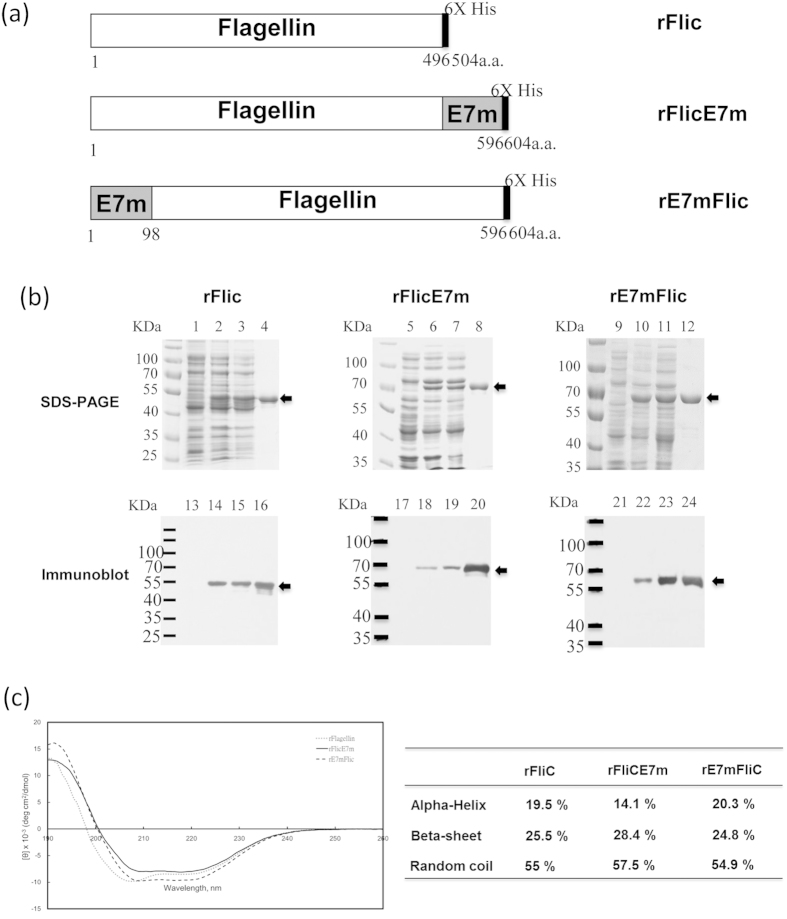
Construction and purification of recombinant proteins. (**a**) The primary structure of the rFliC, rFliCE7m and rE7mFliC proteins are schematically shown; the proteins were expressed in *E. coli* BL21(DE3) star, BL21(DE3) and BL21(DE3), respectively. (**b**) Purification of the rFliC, rFliCE7m and rE7mFliC proteins using 10%, 8% and 8% reducing SDS-PAGE, respectively, followed by Coomassie Blue staining and immunoblotting with anti-His antibodies. Lanes 1–4 show the rFliC purification process; lanes 5–8 show the rFliCE7m purification process; and lanes 9–12 show the rE7mFliC purification process. Lanes 1, 5, and 9: protein expression in the absence of IPTG induction. Lanes 2, 6, and 10: protein expression after IPTG induction. Lanes 3, 7, and 11: cellular extract fractions. Lanes 4, 8, and 12: purified recombinant proteins. Lanes 13–24 show immunoblotting for the purification process; the samples in these lanes are identical to those in lanes 1–12. (**c**) Circular dichroism spectra of rFliC, rFliCE7m and rE7mFliC from 260 to 190 nm were recorded with a 0.1-cm path length quartz cell (left figure). All of the data are reported as the mean residue ellipticity [θ]. The CD spectra were analyzed by the K2D3 program, and the calculated values of the α-helix, β-sheet and random coil are shown on the right.

The flagellin gene from *S. enterica* serovar Typhimurium was optimized for rFliC expression using *Escherichia coli*. The oncogenic site of HPV16 E7 was inactivated by mutation (E7m)^28^, and E7m was fused to the flagellin N- or C-terminus to generate rFliCE7m and rE7mFliC, respectively (Fig. 1a). The proteins were purified from lysates using immobilized metal affinity chromatography (IMAC), evaluated by SDS-PAGE (Fig. 1b, lanes 1–4 are rFliC, lanes 5–8 are rFliCE7m and lanes 8–12 are rE7mFliC) and analyzed by immunoblotting with an anti-His antibody (Fig. 1b, lanes 13–16, lanes 17–20 and lanes 21–24, respectively). The purified proteins were used for functional, immunogenicity and efficacy assays after the removal of lipopolysaccharide (LPS) (less than 0.03 EU/μg).

Next, we examined whether the structure of rFliCE7m and rE7mFliC was altered. We performed far-UV circular dichroism to compare the secondary structures of these preparations. The spectra and the estimated ratios of α-helices, β-sheets and random coils between rFliCE7m and rE7mFliC were slightly different (Fig. 1c), suggesting that the structural differences between rFliCE7m and rE7mFliC might lead to different effects in terms of function, signaling pathway induction or anti-tumor immunity.

### Functional and immunogenicity studies of recombinant flagellin and flagellin fusion proteins

**Figure 2:**
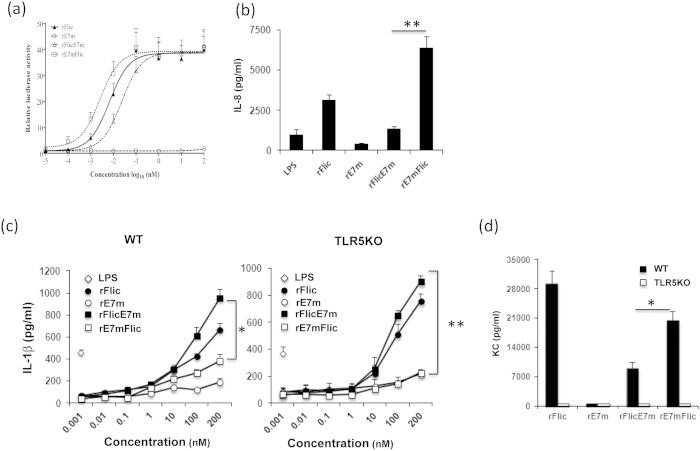
Activation of TLR5 signaling by recombinant proteins *in vitro* and *in vivo*. (**a**) The relative luciferase activity of cell extracts was analyzed using the dual-luciferase reporter assay system. A total of 1.25 × 10^5^ transfected HEK293/hTLR5 cells/well were stimulated with recombinant proteins at the indicated concentrations for 24 hr. The luciferase activities relative to the control (stimulated with medium alone) were measured. (**b**) THP-1 cells were treated with 100 nM of recombinant proteins for 24 hr. Supernatants were collected, and the amount of IL-8 was measured by ELISA. (**c**) WT and TLR5KO BMDCs were seeded at a density of 2 × 10^5^ cells/well. The target protein or LPS (0.1 μg/ml) was added to the LCM medium at the indicated concentration for co-culture with the cells. Cells in medium alone served as the negative control. After 24 hr, the amount of IL-1β in the supernatant was determined. (**d**) KC levels in the sera were measured by ELISA as described in the “Materials and Methods.” All of the data are expressed as the means ± SEM of three independent tests. * and ** indicate p < 0.05 and 0.01, respectively.

Flagellin is capable of activating both the TLR5 and NLRC4/NAIP5 signaling pathways. To determine the TLR5 activity of the recombinant proteins, we performed dual-luciferase reporter assays. Briefly, HEK293 cells expressing human TLR5 (HEK293/hTLR5) were transiently co-transfected with the plasmids pRL-TK and pNF-κB-luc for the expression of a red luciferase and an NF-κB luciferase reporter, respectively. Luciferase expression from the NF-κB reporter mediated by interaction of TLR5 and the recombinant protein was determined (Fig. 2a). The concentrations of rFliC, rFliCE7m and rE7mFliC required for half maximal induction (EC50) were 6.7, 23.7 and 2.6 pM, respectively (Fig. 2a). Interestingly, these values differed significantly, with rE7mFliC most effectively stimulating TLR5 activity. Next, we confirmed these results with another assay. Because the human monocytic cell line THP-1 and several epithelial cell lines secrete IL-8 after TLR5 activation^29^, we chose THP-1 cells as a model to evaluate the TLR5 activity of the recombinant proteins. THP-1 cells were stimulated for 24 hr with 100 nM rFliC, rFliCE7m or rE7mFliC, and IL-8 secretion was measured. Indeed, rE7mFliC induced the highest level of IL-8 secretion, followed by rFliC and rFliCE7m (Fig. 2b), confirming our previous results. Of these recombinant proteins, rE7mFliC activated TLR5 most strongly. To validate our observations *in vivo*, both wild type (WT) and TLR5KO mice were subcutaneously administered 1 nmol rE7m, rFliC, rFliCE7m or rE7mFliC. After 24 hr, sera were collected, and keratinocyte-derived chemokine (KC), IL-6, MCP-1, TNF-α, IL-12p70, IFN-γ and IL-10 concentrations were determined by ELISA. The TNF-α, IL-12p70, IFN-γ and IL-10 levels are lower than the detection limit. The KC, IL-6 and MCP-1 levels were reduced in TLR5KO mouse after flagellin or flagellin fusion proteins stimulation. MCP-1 levels are similar in flagellin or flagellin fusion proteins. Because IL-6 level is very low in sera, it may not suitable for comparison of TLR5 activation (Fig. S1). The administration of rE7mFliC resulted in higher levels of KC compared to rFliCE7m (20465 ± 2179 vs. 9180 ± 1450 pg/ml). Interestingly, administration of rFliC induced a very high level of KC (29187 ± 3134 pg/ml) *in vivo*, suggesting that modification to either end of the protein might interfere with TLR5 binding. Furthermore, KC secretion by the recombinant proteins was abolished after TLR5 was knocked out (Fig. 2c), indicating that rE7mFliC was superior to rFliCE7m at activating TLR5 signaling.

Because flagellin activates not only membrane-bound TLR5 but also the cytosolic NLRC4/NAIP5 inflammasome, it was necessary to determine the effect of the recombinant flagellin fusion proteins on NLRC4/NAIP5 inflammasome signaling. Previous studies have demonstrated that flagellin binds NAIP5 and then interacts with the NLRC4 inflammasome, activating caspase-1 and the induction of IL-1β and IL-18 production^4^,^9^,^12^,^30^. To assess inflammasome activation, the level of IL-1β secretion from bone marrow-derive dendritic cells (BMDCs) was measured^31^. Briefly, BMDCs were cultured in serum-free medium to promote the uptake of recombinant proteins and then stimulated with 20 ng/ml LPS for 2 hr to induce the production of pro-IL-1β. At high doses (100 and 200 nM), rFliCE7m induced the highest level of IL-1β secretion (Fig. 2d). To confirm that the secretion was not caused by TLR5 signaling, the recombinant proteins were incubated with BMDCs isolated from TLR5KO mice. The results showed that the IL-1β secretion levels from the rFliCE7m-treated group remained the highest (Fig. 2d). In contrast to their properties for TLR5 activation, these results suggested that rFliCE7m was more potent for the activation of the NLRC4 inflammasome than rE7mFliC. Accordingly, the IL-1β secretion levels from the rFliCE7m-treated bone marrow-derived macrophage (BMDM) are higher than that of rE7mFlic-treated BMDM (Fig. S2).

### Immunization with rFliCE7m induced higher levels of E7-specific T lymphocyte activity than rE7mFliC

**Figure 3:**
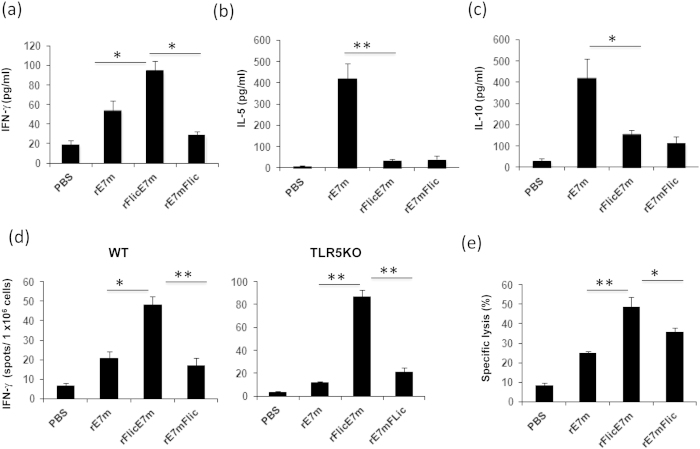
Immunization with rFliCE7m results in a Th1 response and induced higher levels of E7-specific T lymphocyte activity. Immunization of the mice with the indicated recombinant proteins, the isolation of splenocytes and the subsequent stimulation with rE7m were described in the “Materials and Methods.” After stimulation, the supernatants were collected, and the concentration of IFN-γ (**a**) was measured with the TH1/TH2/TH17 cytokine kit. IL-5 (**b**) and IL-10 (**c**) were measured by ELISA. (**d**) Splenocytes (2 × 10^5^ cells/well) were incubated with medium alone or 10 μg/ml of either the RAHYNIVTF (RAH) peptide (from HPV E7) or the SSC peptide (from EBV as a negative control) for 48 hr in an anti-IFN-γ-coated 96-well ELISPOT plate. The IFN-γ-secreting spots were measured using an ELISPOT reader. (**e**) The RAH or control peptide-pulsed splenocytes were labeled with CFSE^hi^ (5 μM) and CFSE^lo^ (0.5 μM) at 37 °C for 10 minutes, respectively. Both cells were resuspended at a density of 2 × 10^7^ cells/ml and mixed at a 1:1 ratio (1 × 10^7^: 1 × 10^7^) in PBS. The mixed target cells were adoptively transferred into immunized mice via tail vein injections. The cells were harvested 18 hr after adoptive transfer and analyzed using FACSCalibur (BD Biosciences, San Jose, CA, USA). To calculate the percentage of specific lysis, the following equation was used: % Specific killing = [1− (%CFSE^hi^/%CFSE^lo^)] × 100. Data are expressed as the means + SEM of three independent tests. * and ** indicate p < 0.05 and 0.01, respectively.

Our data demonstrated that rE7mFliC and rFliCE7m differently induced the TLR5 and NLRC4 signaling pathways. These observations provided us with an opportunity to investigate whether one of these two signaling pathways was more important for the induction of antigen-specific T cell immunity. To assess this, mice were immunized with rE7m, rFliCE7m or rE7mFliC twice at two-week intervals. Splenocytes were isolated and stimulated with rE7m to determine the secretion of IFN-γ and IL-5. The results indicated that immunization with rFliCE7m produced higher levels of the Th1 cytokine IFN-γ (94 ± 10 pg/ml) than immunization with rE7m (54 ± 10 pg/ml) or rE7mFliC (29 ± 4 pg/ml) (Fig. 3a). However, both rFliCE7m and rE7mFliC were less effective at inducing the Th2 cytokines IL-5 and IL-10 (Fig. 3b,c). These results indicated that rFliCE7m might induce Th1-biased immunity. In addition, rFliCE7m also induced higher levels of E7-specific antibody titers than that of rE7mFlic (Fig. S3).

Using an IFN-γ ELISPOT assay and an *in vivo* CTL assay, we examined the cytotoxic T cell immune response. Spleen cells from immunized WT mice were stimulated for 48 hr with the E7 CTL peptide RAHYNIVTF (H-2D^b^-restricted CTL epitope) prior to quantifying IFN-γ-secreting cells. We discovered that rFliCE7m immunization induced significantly higher numbers of IFN-γ-secreting cells compared to the rE7m and rE7mFliC immunizations (Fig. 3d). Similar results were observed in the TLR5KO mice (Fig. 3d), indicating that the TLR5 signaling pathway was not necessary for the induction of CTL activity. Obviously, rFliCE7m was more effective at inducing CTL activity than rE7mFliC, whereas rE7mFliC activated higher levels of TLR5 responses than rFliCE7m. Next, we evaluated E7-specific killing activity in immunized animals and discovered that the E7-specific killing activity of mice immunized with rFliCE7m (49 ± 5%) was higher than that of mice immunized with rE7mFliC (38 ± 2%) or rE7m (25 ± 1%). These results suggested that mice immunized with rFliCE7m elicited stronger CTL responses. Taken together, the results demonstrated that although rE7mFliC was more capable of inducing TLR5 signaling, rFliCE7m was superior at activating NLRC4 signaling and had the capacity to induce stronger antigen-specific T cell immunity.

### Immunization with rFliCE7m induced CD8^+^ T cell-dependent anti-tumor immunity

**Figure 4:**
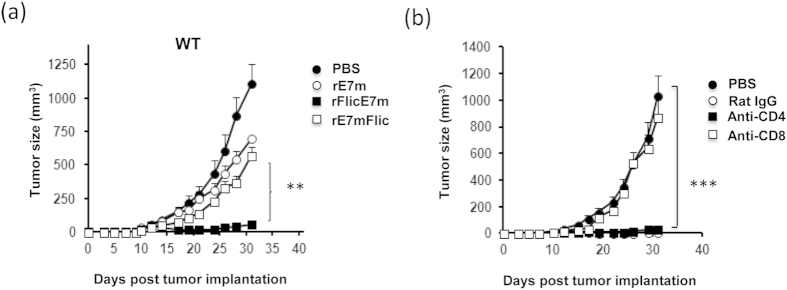
CD8^+^ T cells are necessary for the anti-tumor effects of rFliCE7m. (**a**) WT mice were subcutaneously inoculated with TC-1 cells (2 × 10^5^/mouse) in the abdominal region. After 7 days, the mice were injected with 1 nmol of rE7m, rFliC, rFliCE7m or rE7mFliC, and the control mice were injected with PBS alone. Tumor size was measured two or three times per week. Data are expressed as the means ± SEM of six animals per group. (**b)** Mice were intraperitoneally injected with 0.5 mg of anti-CD4, anti-CD8 or control antibody (Rat IgG) on day 6 after tumor implantation. On day 7, one nmol of rFliCE7m was injected subcutaneously into the right flank. The tumor size was shown as the length × width × width/2 (mm^3^). Data are expressed as the means ± SEM of six mice per group. ** and *** indicate p < 0.01 and 0.001, respectively.

Next, we considered the therapeutic effects of E7mFliC and rFliCE7m in an animal model. C57BL/6 mice were subcutaneously inoculated with 2 × 10^5^ TC-1 cells /mouse. Seven days later, these mice received 1 nmol of recombinant protein through subcutaneous injection. On day 30, tumor sizes were measured, and the values for the PBS, rFliCE7m, rE7mFliC and rE7m treated groups were 1.1 ± 0.15 cm^3^, 0.05 ± 0.01 cm^3^, 0.57 ± 0.06 cm^3^ and 0.69 ± 0.06 cm^3^, respectively (Fig. 4a). To identify which T cell populations (CD4 or CD8) were involved in this anti-tumor immunity, we depleted the subpopulations using anti-CD4 and anti-CD8 antibodies prior to tumor inoculation. As shown in Fig. 4b, tumor growth remained inhibited in the anti-CD4 and rat IgG-treated groups. In contrast, the tumor inhibition effects of rFliCE7m were lost in the CD8-depleted mice.

Altogether, immunization with rFliCE7m elicited strong anti-tumor immunity, whereas rE7mFliC and rE7m were not very effective. The stronger anti-tumor immunity of rFliCE7m was correlated with its ability in induce antigen-specific T cell immunity. Importantly, CD8 T cells were necessary for rFliCE7m-induced anti-tumor immunity.

## Discussion

Flagellin fusion proteins have been shown to induce humoral immunity through the TLR5 and NLRC4 signaling pathways. However, there have been relatively few reports assessing the role of TLR5 or NLRC4 signaling in the development of therapeutic vaccines. Recently, Garaude *et al.* reported that TLR5 and NLRC4/NAIP5 were equally important for the suppression of tumor growth^32^. Here, we showed that the immunogen E7m fused at the N-terminus of flagellin (rE7mFliC) induced relatively higher TLR5 signaling, whereas E7m fused at the C-terminus of flagellin (rFliCE7m) induced stronger NLRC4 inflammasome signaling. Importantly, rFliCE7m but not rE7mFliC induced high levels of T cell immunity and generated an anti-tumor effect. Indeed, rE7mFliC induced E7-specific T cell responses and anti-tumor efficacies that were similar to those of rE7m, which was unable to induce TLR5 signaling. On the other hand, we found that rFlic mixed with rE7m could induce similar levels of anti-tumor activity to rFlicE7m because rFlic preserves both TLR5 and inflammasome activity (data not show). Therefore, our data clearly demonstrated that activation of TLR5 signaling might not be sufficient for the induction of cellular immunity by flagellin-adjuvanted antigens, and NLRC4 signaling might be more relevant for these cellular processes. Conceptually, this conclusion is important for the development of flagellin-based therapeutic vaccines.

Over the past decade, a number of reports have described the adjuvant property of flagellin in the context of a broad range of recombinant protein vaccines^18^,^33^,^34^,^35^,^36^,^37^. However, a number of unknown factors remain to be elucidated. Flagellin and the antigen could be co-administered separately or as a fusion protein; however, immunization with fusion antigens markedly enhanced protective immune responses compared to mixtures of flagellin and antigen^26^. This effect might be due to the increased efficiency achieved by co-delivery of the adjuvant and antigen to the same TLR5^+^ antigen presenting cells using flagellin fusion proteins^18^. However, whether TLR5 signaling is necessary to enhance antigen-specific immune responses is controversial. Instead, the activation of TLR5 has been shown to be important for the induction of protective immunity^20^,^27^,^38^,^39^, and flagellin has been reported to induce protective immunity through a TLR5- and MyD88-independent pathway^5^,^15^,^39^. Using a flagellin-expressed cancer vaccine, Johan Garaude *et al.* discovered that both TLR5 and NLRC4 were equally important for the induction of CD4^+^ and CD8^+^ T cell responses^32^. Here, we demonstrated that NLRC4 contributes to the induction of anti-tumor immunity and that TLR5 might play a role transporting flagellin fusion antigens into cells to activate cytosolic NLRC4 inflammasome signaling. Then, the endocytic antigen could be further processed and presented to both CD4^+^ and CD8 T^+^ cells. This hypothesis is in agreement with the results reported by Letran *et al.* and Sanders *et al.*, who demonstrated that the induction of adaptive immunity by flagellin did not require the activation of TLR5/MyD88 signaling^5^. Letran *et al.* found that TLR5 was an endocytic receptor that enhanced flagellin-specific CD4^+^ T cell responses through a MyD88-independent pathway^40^. Furthermore, Bates *et al.* proposed that flagellin promoted CD8^+^ T cell activation in a manner that was independent of its TLR5 signaling activity and acted through an unknown mechanism to facilitate antigen processing^16^. Therefore, we suggest that flagellin fusion proteins need to be modified to induce strong NLRC4 inflammasome signaling and concomitantly preserve TLR5 binding activity for the induction of CD8^+^ T cell responses.

Although both the C and N terminal regions of flagellin are necessary for TLR5 activation, only the N-terminal region is needed for the activation of the NLRC4 inflammasome. Therefore, the fusion of an antigen to the terminus of flagellin might change its bioactivity^23^,^24^. To maintain flagellin bioactivity and antigen conformation, Liu *et al.* fused the hemagglutinin antigen (HA1) of H1N1 to the C-terminus of flagellin (STF2.HA1) or replaced the D3 domain of flagellin with one or two copies of HA1 to construct STF2R3.HA1 and STF2R3.2xHA1, respectively. The authors discovered that the relative bioactivity ranking of these fusion proteins was STF2R3.HA1 > STF2R3.2xHA1 > STF2.HA1^40^. Another study showed that fusion of the fimH protein to the N-terminus of flagellin (FimH.FliC) retained a level of TLR5 activity similar to FliC^26^. Moreover, antigen fused to the C-terminus of D2-D3-deleted flagellin (rflaA.Ova) significantly reduced TLR5 activity 10-fold compared to rflaA. This reduction in TLR5 activity was not due to differences in the secondary structures of rflaA and rflaA.Ova because the CD spectra appeared to be similar, if not identical. We analyzed the CD spectra of rFliC, rFliCE7m and rE7mFliC and found that the spectra and the calculated ratios of α-helices, β-sheets and random coils were slightly different between rFliCE7m and rE7mFliC. However, further studies are required to reveal the mechanism underlying these differences and their contribution to the bioactivity of flagellin fusion proteins. Here, we demonstrated that fusion of E7m to different termini of flagellin resulted in different TLR5 and NLRC4 induction activities. Fusion of rE7m to the N-terminus of flagellin resulted in better induction of TLR5 activity. Conversely, rFliCE7m with a free flagellin N-terminus retained NLRC4 activity, whereas this activity was lost in rE7mFliC. However, whether this phenomenon is universal or restricted to a small number of antigens (including E7m) remains to be determined.

A number of biological fusion proteins containing TLR agonist activity have been developed as vaccine candidates, including a recombinant lipoprotein with TLR2 agonist activity and a recombinant flagellin fusion protein with TLR5 or NLRC4 activity^18^,^41^. These recombinant proteins showed differences from the parental protein in TLR activity. Our previous data showed that the recombinant lipoprotein rlipo-D1E3 activated the same TLR2 signaling pathway as a synthetic lipopeptide but induced differential cytokine production^42^. Similarly, in this report we demonstrated that rFliCE7m and rE7mFliC showed different capacities to activate TLR5 and NLRC4 inflammasome signaling. These findings should be considered in the rational design of recombinant fusion proteins for use as vaccine candidates.

## Methods

### Cell lines

TC-1, a mouse epithelial cell line transformed with the oncogenes Ras, HPV16 E6 and E7, was a kind gift from Dr. TC Wu (Johns Hopkins University). TC-1 cells were cultured in LCM medium (RPMI 1640 supplemented with 10% (v/v) heat-inactivated fetal bovine serum, 50 nM sodium pyruvate, 20 mM HEPES, 100 units/ml penicillin, 100 μg/ml streptomycin sulfate and 50 nM β-mercaptoethanol). The human monocytic cell line THP-1 was maintained in RPMI 1640 medium containing 10% FBS. 293-hTLR5 cells, which were generated by transfecting HEK293 cells with the human TLR5 gene, were purchased from InvivoGen and maintained in DMEM (GIBCO-BRL, Grand Island, NY, USA) supplemented with 10% heat-inactivated fetal bovine serum, penicillin (100 units/ml), streptomycin (100 μg/ml), and blasticidin (10 μg/ml).

### Reagents

All of the chemicals were purchased from Sigma (St. Louis, MO, USA) and Merck (Darmstadt, Germany). Restriction enzymes and DNA ligase were purchased from New England Biolabs, Inc. (Beverly, MA, USA). The primers used for cloning were purchased from Mission Biotech, Inc. (Taipei, Taiwan). Mouse recombinant GM-CSF was purchased from Peprotech, and lipopolysaccharide (LPS; *E*. *coli* endotoxin serotype 055:B5) was purchased from Sigma-Aldrich. Carboxyfluorescein diacetate succinimidyl ester (CFSE) was purchased from Invitrogen.

### Cloning, expression and purification of recombinant proteins

The expression vector encoding the inactive E7 gene (E7m) and the expression of recombinant proteins in the *E. coli* system were described in our previous study^28^. A similar protocol was used to make the constructs. Briefly, the FliC sequence from *S. typhimurium* (accession number: WP_000079805) was codon-optimized using the table of codon usage in *E. coli* and synthesized by MDBio, Inc. (Taiwan). Based on the gene sequences of E7m and FliC, six primers were designed to clone the plasmids (Table 1). Three plasmids (pFliC, pFliCE7m and pE7mFliC) were constructed in the expression vector pET-22b (+) (Novagen, Madison, WI, USA) for the expression of the recombinant proteins rFliC, rFliCE7m and rE7mFlic, respectively. As a result, the C-terminus of these recombinant proteins contained an additional hexahistidine tag (HisTag). The *E. coli* strains BL21(DE3) star, BL21(DE3) and BL21(DE3) (Invitrogen, Carlsbad, CA, USA) were transformed with the plasmids pFliC, pFliCE7m and pE7mFliC, respectively. Next, the recombinant proteins were induced by adding 1 mM IPTG for 4 hr, and the cells were harvested by centrifugation. The purification process of rFliC, rFliCE7 and rE7mFliC was similar to that described in our previous study for rE7m purification^28^. After reducing the LPS levels to less than 0.03 EU/μg, the purified rFliC, rFliCE7 and rE7mFliC proteins were analyzed by SDS-PAGE and immunoblotting and verified by N-terminal amino acid sequencing.

### Circular dichroism (CD) spectrometry

We followed Greenfield’s protocol to obtain the CD spectra^43^. Briefly, lyophilized rFliC, rFliCE7m, and rE7mFliC proteins were reconstituted in distilled water, dialyzed for 16 hr in distilled water, and diluted to the optimal concentration (0.51, 0.22 and 0.35 mg/ml, respectively). CD spectra in the far-UV region (190 to 260 nm) were detected using a J-815 spectropolarimeter (Jasco Inc., Easton, MD, USA) with a 0.1-mm path length cell. Each spectrum was scanned with a 0.5-nm bandwidth. The spectra were visualized with Spectra Manager software (Jasco Inc., Easton, MD, USA), and the CD spectra were analyzed by the K2D3 program^44^.

### NF-κB luciferase reporter assay

HEK293/hTLR5 cells (InvivoGen San Diego, CA, USA) were plated onto 24-well plates (1.25 × 10^5^ cells/well) and transiently co-transfected with 0.02 μg of pNF-κB-Luc and 0.02 μg of the pRL-TK internal control plasmid (Promega, Madison, WI, USA) using Lipofectamine 2000 (Invitrogen, Carlsbad, CA, USA). After 24 hr of incubation, the transfected cells were stimulated with the indicated concentration of recombinant proteins for an additional 24 hr. The cells were lysed, and the luciferase activity was measured by a dual-luciferase reporter assay system (Promega Co., Madison, WI, USA). Both firefly (NF-κB) and Renilla (TK) luciferase activities were detected with a Berthold Orion II luminometer (Pforzheim, Germany).

### Cytokine release upon stimulation with recombinant proteins

Female 6–8-week-old C57BL/6 mice were immunized twice subcutaneously with 1 nmol rE7m, rFliCE7m or E7mFliC at 14-day intervals. On day 7 after the second immunization, the mice were sacrificed, and splenocytes were harvested and stimulated with rE7m (10 μg/ml) for 4 days. The supernatants were collected, and IL-5 production was analyzed using an ELISA kit (eBioscience San Diego, CA, USA). The production of IFN-γ and IL-10 was analyzed using a cytometric bead array using the mouse Th1/Th2/Th17 cytokine kit and detected with a FACSarray flow cytometer (Becton Dickinson Immunocytometry Systems, San Jose, CA, USA). To examine IL-8 secretion, THP-1 cells (2 × 10^5^ cells) were stimulated with LPS (100 ng/ml) or 100 nM of the indicated recombinant proteins for 24 hr in THP1 medium. The concentration of the cytokine IL-8 secreted by THP-1 cells was determined using an ELISA kit (eBioscience San Diego, CA, USA). To induce keratinocyte chemoattractant (KC), WT or TLR5KO mice were immunized with a subcutaneous injection of 1 nmol rE7m, rFliCE7m or E7mFliC. One day later, the mice were bled via the retrobulbar intraorbital capillary plexus. KC levels in the serum were measured with an ELISA kit (RayBiotech, Inc., Norcross, GA, USA).

### ELISPOT assay

IFN-γ-secreting cells were analyzed using an IFN-γ ELISPOT assay as previously described^28^. Briefly, splenocytes (5 × 10^5^/well) were added to 96-well microplates (MSIP, Millipore) coated with anti-mouse IFN-γ capture antibodies (clone AN18) and incubated with 10 μg/ml of the indicated peptides in lymphocyte culture medium (LCM, RPMI 1640 medium supplemented with 10% (v/v) fetal bovine serum, 50 units/ml penicillin/streptomycin, 20 mM HEPES, and 0.5 μM β-mercaptoethanol) for 48 hr. The peptides RAHYNIVTF (RAH, E7_49–57_) and SSCSSCPLSKI (SSC, LMP2_340–350_) represented the CTL epitopes of the HPV16 E7 oncoprotein and EBV latent membrane protein 2, respectively. After incubation, the cells were removed by washing the plates with 0.05% (w/v) Tween-20 in PBS. A 100 μl aliquot containing 10 mg/ml of a biotinylated anti-IFN-γ detection antibody (clone R46A2, eBioscience San Diego, CA, USA) was added to each well and incubated for 2 hr. The spots were developed using 3-amine-9-ethyl carbazole (AEC substrate) (Sigma, St. Louis, MO, USA) and counted by an ELISPOT reader (Cellular Technology Ltd., Shaker Heights, OH, USA).

### Culture of bone marrow-derived macrophages and dendritic cells

Bone marrow-derived macrophages (BMDMs) from wild-type or TLR5KO mice were cultured as previously described^45^. Briefly, mouse bone marrow cells were cultured at a density of 1 × 10^6^ cells/ml in 24-well plates with 1 ml of LCM in the presence of 20% L929-conditioned medium. On day 3, an additional 1 ml of LCM containing 20% L929-conditioned medium was added. On day 7, the cells were washed 2 times with PBS and 1 ml of LCM was added; then, the cells were incubated at 37 °C overnight. For BMDC cultures, 2 × 10^6^ bone marrow cells were seeded into a 90 × 15 mm Petri dish (α-Plus) with 10 ml of LCM in the presence of 20 ng/ml mouse GM-CSF (PeproTech). The cells were incubated at 37 °C with 5% CO_2_ for 3 days; then, another 10 ml of LCM containing 20 ng/ml GM-CSF was added to the cell culture. The floating DCs were harvested for experiments on day 6.

Prior to stimulation with the recombinant flagellin fusion proteins, LPS (20 ng/ml) was added to the BMDM or BMDC culture for 2 hr to induce pro-IL-1β; then, the cells were washed two times with RPMI 1640 medium supplemented with 100 units/ml penicillin and 100 mg/ml streptomycin sulfate. A density of 1 × 10^6^ BMDM cells/well or 2 × 10^5^ BM-DC cells/well was incubated with the indicated concentrations of rE7m, rFliC, rFliCE7m or rE7mFliC for 24 hr. The secretion of the cytokine IL-1β by BMDCs or BMDM was determined using an ELISA kit (eBioscience San Diego, CA, USA).

### Mice and tumor challenge

Female 6- to 12-week-old mice were obtained from the National Laboratory Animal Breeding and Research Center (Taipei, Taiwan). All of the animals were housed at the Animal Center of the National Health Research Institutes (NHRI) and maintained in accordance with the institutional animal care protocol. All of the animal studies were conducted in accordance with the protocol approved by the Institutional Animal Care and Use Committee of the NHRI (approval ID: NHRI- IACUC-097077-A). For the tumor therapy experiment, WT and TLR5KO C57BL/6 mice were injected with 2 × 10^5^ TC-1 tumor cells. Then, 1 nmol of rE7m, rFliCE7m, rE7mFliC or PBS was administered to the WT or TLR5 KO C57BL/6 mice 7 days after tumor implantation. Tumor diameters were measured in two orthogonal dimensions using a caliper two or three times per week. Tumor volumes were calculated from the measurements according to the following formula: (length  ×  width^2^)/2.

### Depletion of CD4^+^ or CD8^+^ T lymphocytes in mice

The T cell-depletion experiments were described previously^46^. Briefly, groups of mice were treated intraperitoneally (i.p.) with either 0.5 mg of a mouse anti-CD4 antibody (clone GK1.5, eBioscience) or a mouse anti-CD8 antibody (clone 53-6.7, eBioscience) to deplete CD4^+^ or CD8^+^ T lymphocytes, respectively. Additionally, 0.5 mg of rat IgG (Sigma, Deisenhofen, Germany) was administered to the control group. The efficiency of depletion was greater than 90% as determined by flow cytometry using different clones of fluorescence-conjugated anti-CD4 or anti-CD8 antibodies. The mice were implanted with 2 × 10^5^ TC-1 tumor cells and injected with the depletion antibodies one day prior to immunization with rFliCE7m. On the seventh day after tumor inoculation, 1 nmol of rFliCE7m was administered to the C57BL/6 mice (six per group).

### *In vivo* cytolytic activity assay

To examine the antigen-specific cytolytic activity of the immunized mice *in vivo*, peptide-pulsed syngenic splenocytes were used as target cells for the killing assay. RBC-lysed splenocytes were counted and divided into two equal portions, and cells at a density of 2 × 10^7^/ml were incubated with a specific peptide (HPV16 E7_49–57_) or non-specific peptide (OVA_257–264_) at 37 °C for 30 minutes. To detect differences between the peptide-pulsed target cells, cells from the two groups were labeled with CFSE^hi^ (5 μM) and CFSE^lo^ (0.5 μM) at 37 °C for 10 minutes, respectively. Both groups of cells were resuspended at a density of 2 × 10^7^/ml, and the cells were mixed in a 1:1 ratio (1 × 10^7^: 1 × 10^7^) in PBS. The mixed target cells were adoptively transferred into immunized mice via tail vein injection 7 days after immunization. The experimental cells were harvested 18 hr after adoptive transfer and analyzed using a FACSCalibur (BD Biosciences, San Jose, CA, USA). To calculate the percentage of specific lysis, the following equation was used: % Specific killing = [1− (%CFSE^hi^/%CFSE^lo^)] × 100.

### Statistical Analysis

Tumor growth kinetic experiments were analyzed using a two-sided unpaired Student’s *t* test when comparing groups by specific time points. All of the other cell experiments were analyzed by Student’s *t*-test. For all the results, p < 0.05 was considered to be statistically significant.

## Additional Information

**How to cite this article**: Lin, K.-H. *et al.* Carboxyl-terminal fusion of E7 into Flagellin shifts TLR5 activation to NLRC4/NAIP5 activation and induces TLR5-independent anti-tumor immunity. *Sci. Rep.* **6**, 24199; doi: 10.1038/srep24199 (2016).

## Supplementary Material

Supplementary Information

## Figures and Tables

**Table 1:** The primer sequences used in this study.

Names	Sequences
FliCN-F	5′-GGAATTCCATATGGCGCAGGTGATTAACACCAACA-3′
FliCX-R	5′-CCGCTCGAGACGCAGCAGGCTCAGCACGTTC-3′
FliCB-R	5′-CGCGGATCCACGCAGCAGGCTCAGCACGTTC-3′
FliCB-F	5′-CGCGGATCCATGGCGCAGGTGATTAACACCAACA-3′
E7mB-R	5′-CGCGGATCCCGGTTTCTGGCTCGCAATCG-3′
E7mN-F	5′-GGAATTCCATATGCATGGCGATACCCCGACCCT-3′

The introduced restriction sites are underlined.
