# Supplementary material for: Carboxyl-terminal fusion of E7 into Flagellin shifts TLR5 activation to NLRC4/NAIP5 activation and induces TLR5-independent anti-tumor immunity
Source: Sci Rep. 2016 Apr 11;6:24199. doi: 10.1038/srep24199 (PMC4827055; doi:10.1038/srep24199)
Supplement: Supplementary Information [file srep24199-s1.pdf]

Carboxyl-terminal fusion of E7 into Flagellin shifts TLR5 activation to NLRC4/NAIP5 activation and induces TLR5-independent anti-tumor immunity

Authors: Kuo-Hsing Lin[1,a], Li-Sheng Chang[1,4,a], Chun-Yuan Tian[1], Yi-Chen Yeh[1], Yu-Jie Chen[1,4], Tsung-Hsien Chuang[2], Shih-Jen Liu[1,3,*], Chih-Hsiang Leng[1,3,*]

Supplemental Figure 1

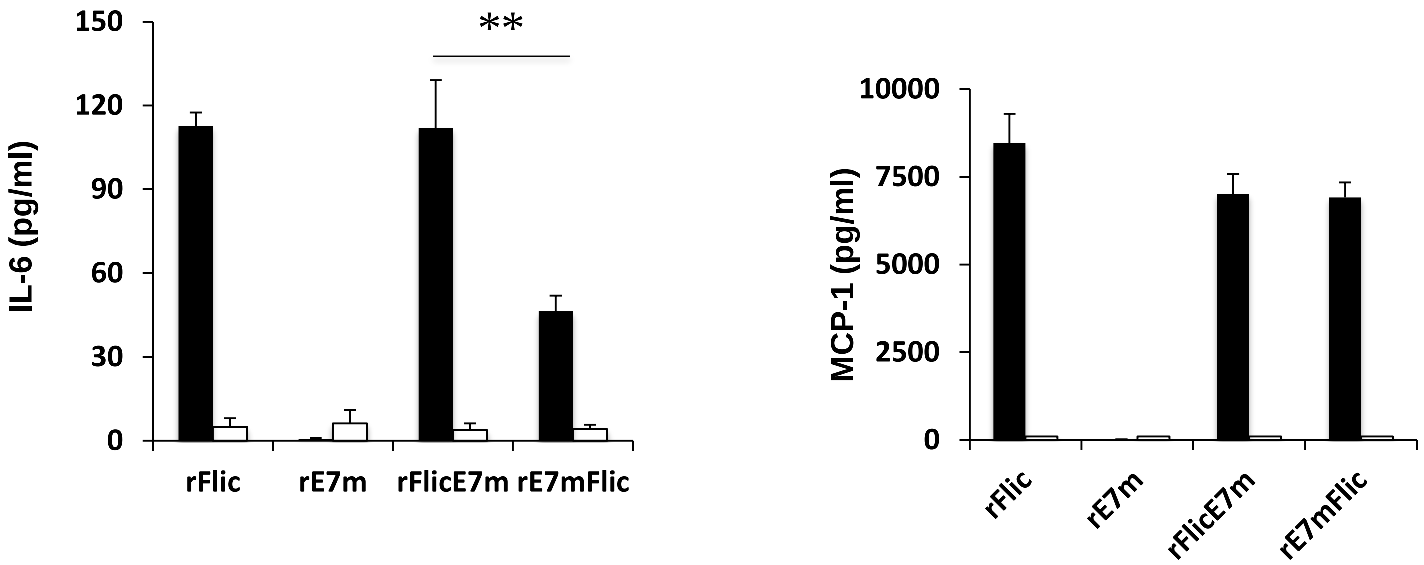

Figure S1. Activation of TLR5 signaling by recombinant proteins *in vivo*. IL-6 and MCP-1 levels in the sera were measured by ELISA as described in the “Materials and Methods.” All of the data are expressed as the means ± SEM of three independent tests. * and ** indicate $p < 0.05$ and 0.01, respectively.

Supplemental Figure 2

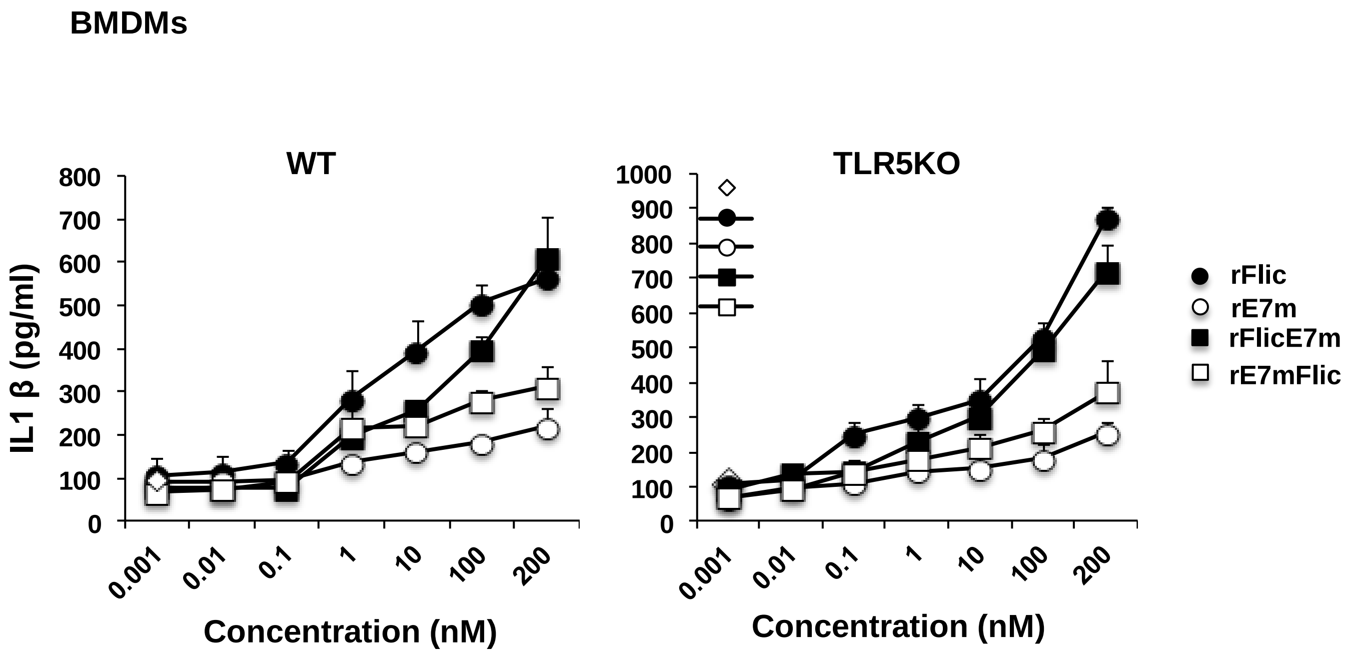

Figure S2. NLRC4 inflammasome activation by flagellin fusion proteins. BMDMs from WT or TLR5KO mice were seeded at a density of 1x10^6 cells/well in serum-free medium. The recombinant proteins (0.001-20 nM) or LPS (0.1 μg/ml) were added to each well and incubated for 24 hr. Supernatant was collected for the IL-1β ELISA. Data are expressed as the means + SEM of three independent experiments.

Supplemental Figure 3

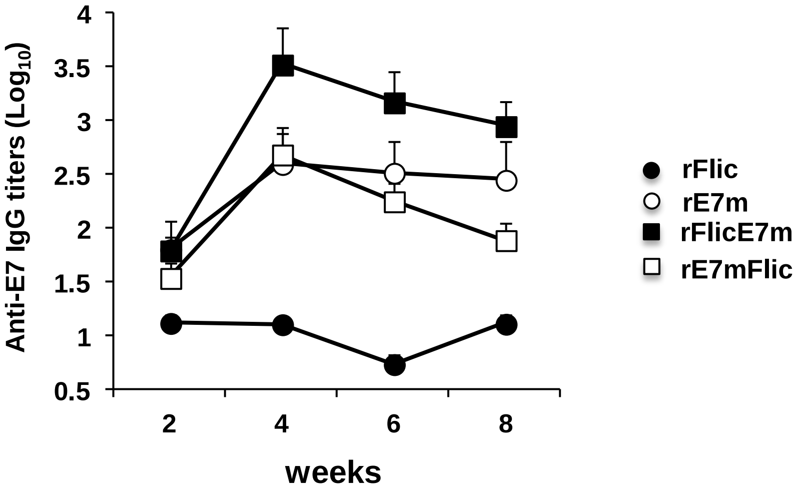

Figure S3. Immunization with rFlicE7m induced higher levels of anti-E7 antibody responses than rE7mFlic. Mice were immunized with 1 nmol of rFlic, rE7m, rFlicE7m or rE7mFlic twice at two-week intervals. Sera were collected at weeks 0, 2, 4, 6, and 8, and the anti-E7 IgG antibody titer was analyzed by ELISA. Data are shown as the means + SD.
